# Structure sensitive photocatalytic reduction of nitroarenes over TiO_**2**_

**DOI:** 10.1038/s41598-017-08599-2

**Published:** 2017-08-18

**Authors:** Swapna Challagulla, Kartick Tarafder, Ramakrishnan Ganesan, Sounak Roy

**Affiliations:** 1 Department of Chemistry, Birla Institute of Technology and Science (BITS) Pilani, Hyderabad Campus, Jawahar Nagar, Shameerpet Mandal, Hyderabad, 500078 India; 2 Department of Physics, National Institute of Technology Karnataka, Surathkal, Mangalore, 575 025 Karnataka India

## Abstract

It is a subject of exploration whether the phase pure anatase or rutile TiO_2_ or the band alignment due to the heterojunctions in the two polymorphs of TiO_2_ plays the determining role in efficacy of a photocatalytic reaction. In this work, the phase pure anatase and rutile TiO_2_ have been explored for photocatalytic nitroarenes reduction to understand the role of surface structures and band alignment towards the reduction mechanism. The conduction band of synthesized anatase TiO_2_ has been found to be more populated with electrons of higher energy than that of synthesized rutile. This has given the anatase an edge towards photocatalytic reduction of nitroarenes over rutile TiO_2_. The other factors like adsorption of the reactants and the proton generation did not play any decisive role in catalytic efficacy.

## Introduction

Understanding the relationship between the surface structure of solid materials and their catalytic reactivity has wide interest and applications in surface science and catalysis. Unlike dark-catalysis, reaction sensitivity to the structure in photocatalysis is not only limited to the efficiency of adsorption of reactants over the particular surface, but also influenced by factors like generation of photoelectrons, their diffusion to the surface, electron-hole recombination, position of conduction band and transfer of the conduction band electrons to the reactants etc. TiO_2_ is the most widely used photocatalyst because of its chemically stable and biologically benign nature^1–4^. The two polymorphic crystal structures of TiO_2_, anatase and rutile are commonly used in photocatalysis, while pure brookite is cumbersome to prepare, and amorphous TiO_2_ has no photocatalytic activity^5^. Not only the anatase TiO_2_ is believed to be a better photocatalytic material than rutile^6^, but also the different crystallographic orientations of the same material exhibit different activities^7–10^. However, the lacuna on explaining the structure sensitive TiO_2_ reactivity is still a topic of debate. The structure sensitivity can be explained crystallographically as anatase TiO_2_ is constituted with about 90% exposed (101) and rutile with (110) facets. The difference in efficiency of photocatalysis between rutile and anatase could be due to these different exposed surfaces^11–15^. The bandgap (E_g_) of TiO_2_ is also crucially responsible for its electronic structure and E_g_ of rutile is slightly smaller than that of anatase TiO_2_^16, 17^. The general consensus over the conduction band position of the two polymorphs are arguable as the electrochemical impedance analyses established the conduction band of anatase lies 0.2 eV above that of rutile^18–21^ while, the photoemission measurements have reported the reverse trend^22–24^. This band alignment, would play a significant role on transferring the photogenerated electrons from anatase and rutile to the reactants. Despite the intensive study of TiO_2_, there is no generally accepted explanation for the differences of photocatalytic activity or mechanism of different polymorphs or surface orientations.

Therefore, we took up this project to probe the role of different polymorphic structures of TiO_2_ over a well-regarded photocatalytic reaction: abatement of nitroarenes. The nitroarenes are extensively used in the chemical industries as explosives, pesticides, solvents etc., and are also generated from the production, storage, and demilitarization of munitions^25–28^. The nitroarenes are carcinogenic, genotoxic, endocrine disruptor chemicals to human beings and also responsible for diseases like methaemoglobinaemia^29^. Due to these various noxious effects of nitroarenes, many researchers are prompted to focus on photocatalytic abatement of nitroarenes by TiO_2_^27, 28, 30–32^. The reduction of nitroarenes would valorize the harmful pollutants into important industrial intermediates, as functionalized aminoarenes are widely used for the synthesis of dyes, fine chemicals, agrochemicals, and pharmaceuticals^33–37^.

Nitroarenes photoreduction over TiO_2_ is a 6-electron process, which utilizes the photoexcited electrons from the conduction band and protons from a suitable source. The overall stoichiometric reaction can be expressed as follows: $Ar-N{O}_{2}+6{H}^{+}+6{e}_{CB}^{-}\to Ar-N{H}_{2}+2{H}_{2}O$. TiO_2_ polymorphs with different exposed facets would adsorb the nitroarenes with different magnitudes at optimized orientation^23, 38^. The photogenerated electrons in the conduction band would move downhill to the available molecular orbitals of nitroarenes for the smooth reduction reaction^39–41^. Therefore, the adsorption of nitroarenes, the position of conduction band minima with respect to the reduction potential of nitroarenes, number of photogenerated electrons over the catalysts, rate of electron-hole recombination, and proton transfer are the crucial parameters to introspect the mechanistic details of nitroarenes photoreduction over a particular exposed facet of TiO_2_. Here, we report structure sensitive photocatalytic reduction of nitroarenes over two polymorphs of TiO_2_ semiconductors. Phase pure anatase was synthesized by solution combustion method, whereas, rutile was synthesized by polymerizable sol-gel route. Both the catalysts were structurally, morphologically and electronically characterized before studying their photocatalytic performances of nitroarenes reduction. The catalytic mechanism was understood from the surface structure and the electronic band structure.

## Methods

### Synthesis and characterization

Anatase TiO_2_ was synthesized by the low temperature initiated, self-propagating, single step solution combustion method using glycine as fuel^42–44^. As a Ti-precursor, TiO(NO_3_)_2_ was prepared by hydrolyzing titanium (IV) isopropoxide followed by digesting with conc. HNO_3_. The oxidizing valency of TiO(NO_3_)_2_ (−10) was stoichiometrically balanced with the total reducing valency of glycine (+9) to release the maximum energy. Therefore, in a typical synthesis, 1 g of TiO(NO_3_)_2_ solution was mixed with 0.442 g of glycine in a 300 mL borosilicate dish and combusted at 450 °C in a pre-heated muffle furnace. The solution boiled with frothing and foaming with concomitant dehydration. At the point of complete dehydration, the redox mixture ignites yielding a voluminous finely dispersed solid product. After complete combustion, the obtained anatase TiO_2_ powders were scraped off and grounded using mortar and pestle. The rutile TiO_2_ was synthesized by the polymerizable sol–gel method^45–48^. In a typical synthesis, 1.92 g of titanium (IV) isopropoxide was mixed with 2.34 mL of methacrylic acid in 1:4 molar ratio in an inert atmosphere to obtain methacrylate substituted titanium complex. To this, 0.5 mL of 10% solution of benzoyl peroxide in acetone was added to initiate the free radical polymerization. The resultant mixture was polymerized at 125 °C for 1 h and finally calcined at 550 °C for 4 h to obtain rutile TiO_2_.

The synthesized catalysts were structurally characterized with Rigaku Ultima IV X-ray diffractometer using Cu K_α_ source at a scan rate of 1° min^−1^. UniRAM 3300 Raman microscope with an incident laser wavelength of 532 nm was used to measure the Raman spectra of the synthesized catalysts. The surface morphology of the synthesized catalysts were studied with field emission scanning electron microscope (FE-SEM, Carl-Zeiss ULTRA-55). The valance band edges of TiO_2_ in both anatase and rutile were measured using X-ray photoelectron spectrometer (XPS, FEI, PHI 5000 Versa Prob II) with Al K_α_ radiation. The band gap of anatase and rutile TiO_2_ was measured with the help of JASCO V-670 UV-visible spectrophotometer. The current generation as a function of applied voltage under UV light exposure was measured by Keithley 2450 source measure unit.

### Adsorption and catalysis

To study the adsorption isotherm, batch adsorption experiments were carried out in dark with both the catalysts, where the catalyst amount was kept constant at 10 mg, and 20 mL of 2, 4, 6, 8 and 10 mg/L of nitroarenes were used. The solutions were stirred in dark for 2 h and the aliquots were drawn in regular time interval, centrifuged and the concentrations were measured with UV-visible spectrophotometer (JASCO V-650). The obtained data were attempted to fit with Langmuir and Freundlich isotherms. First-principles density functional theory (DFT) calculations were also performed to investigate the adsorption of nitroarenes on both TiO_2_ surfaces using the full-potential Vienna ab-initio simulation package (VASP)^49, 50^. The applied exchange–correlation functional was the GGA in the Perdew and Wang (PW91) parametrization^51^. Projector augmented wave potentials were used^52, 53^ and the wave functions were expanded in the plane wave basis with a kinetic energy cutoff of 500 eV. In the simulations, the forces on each of the atoms were calculated using the Hellmann-Feynman theorem, and were subsequently used to perform a conjugate gradient structural relaxation. The structural optimizations were continued until the forces on the atoms converged to less than 1 meV/Å. This optimization has been completely carried through for all combinations of nitroarenes absorbed on anatase and rutile TiO_2_ surfaces.

A cylindrical shaped batch photoreactor fitted with a 125 W medium pressure mercury vapor lamp of maximum intensity of emission at 365 nm (from SAIC India Ltd) was used for the photocatalytic reduction of nitroarenes in aerobic and anaerobic conditions. Water was circulated around the lamp to prevent the IR radiation and also to maintain the reaction at constant room temperature. For the photoreductions, 50 mg of TiO_2_ catalyst was added to 50 ppm of nitroarenes in a 100 mL solvent mixture of 75:25 water-methanol. The reaction mixture was stirred in dark for 30 min to reach the adsorption-desorption equilibrium before illumination of the light. After the onset of the photoreaction, 2 mL of aliquot was collected in regular time intervals and the reaction progress was monitored by UV-visible spectrophotometer (JASCO V-650) and gas chromatograph (Shimadzu GC 2010 plus) equipped with EB-1 column and FID detector. The hydrogen production over the catalysts was also performed in the same batch photoreactor and was monitored using online gas chromatograph (DGA I, Mayura Analytical Private Limited, India) equipped with Haysep-A and Molecular Sieve columns and TCD detector.

Electrocatalytic reductions of nitrobenzene were performed by cyclic voltammetry (CV) with a computer controlled potentiostat (AUTOLAB PGSTAT302N, Metrohm Autolab B.V). A conventional three-electrode arrangement was employed, a platinum electrode as a counter electrode, an Ag/AgCl electrode as a reference electrode, and the working electrode was made by pelletizing 100 mg of anatase and rutile TiO_2_ with 30 wt % of graphitic carbon in a customized glass jacket. Na_2_SO_4_ (0.1 M) in a 100 mL solvent mixture of 75:25 water-methanol containing 100 mg/L of nitrobenzene were used as electrolyte. Argon was purged for 20 min to remove the dissolved oxygen and all the experiments were carried out at room temperature. The potential was varied from 0.5 V to −2.0 V at a scan rate of 20 mV/s to measure the reduction potential of nitrobenzene by cyclic voltammetry method.

## Results and Discussion

**Figure 1:**
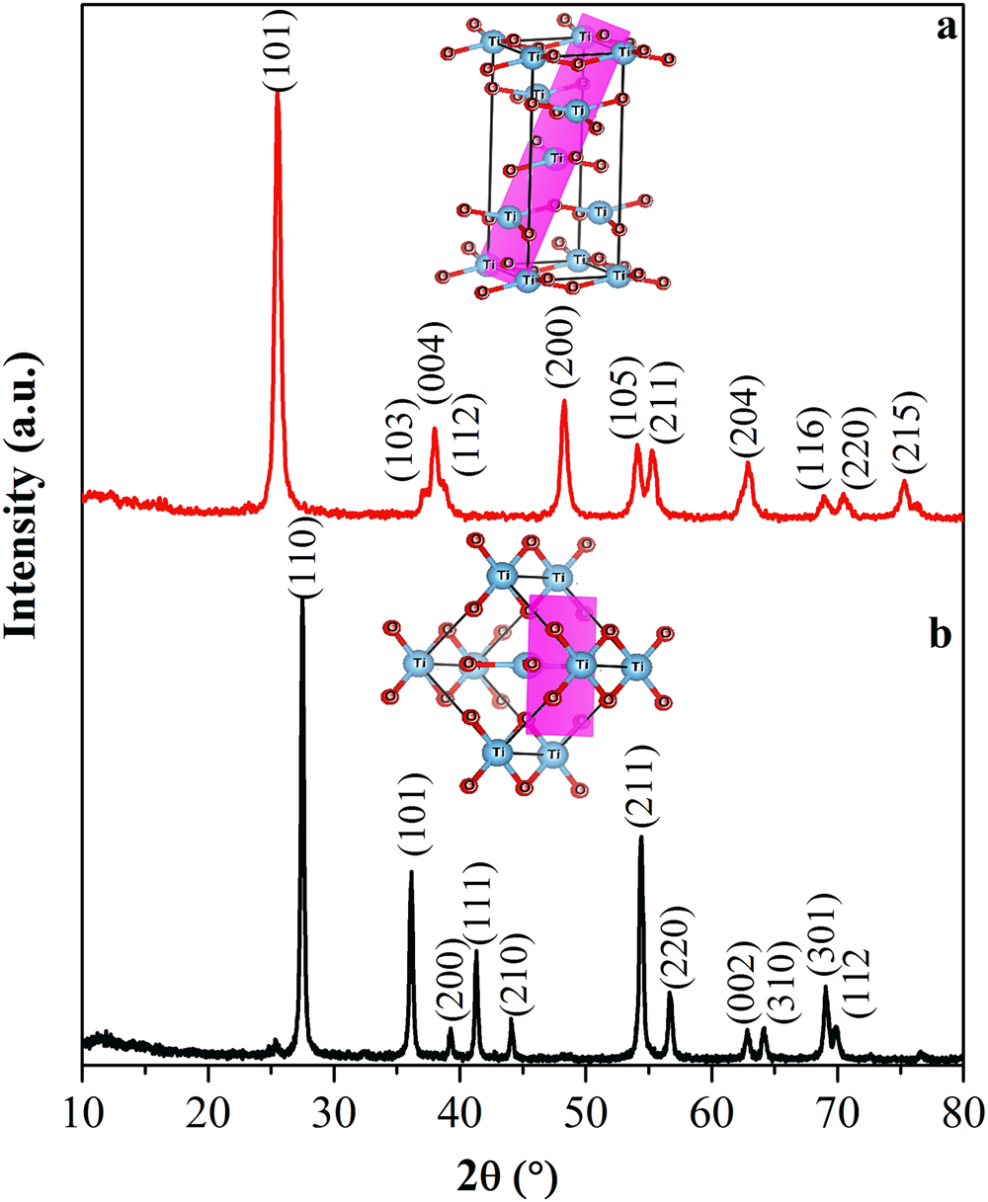
XRD patterns of (**a**) anatase, and (**b**) rutile TiO_2_. The unit cells are drawn in inset with the lattice parameters obtained from Rietveld refinement. The major reflecting plane of each polymorph is highlighted.

**Figure 2:**
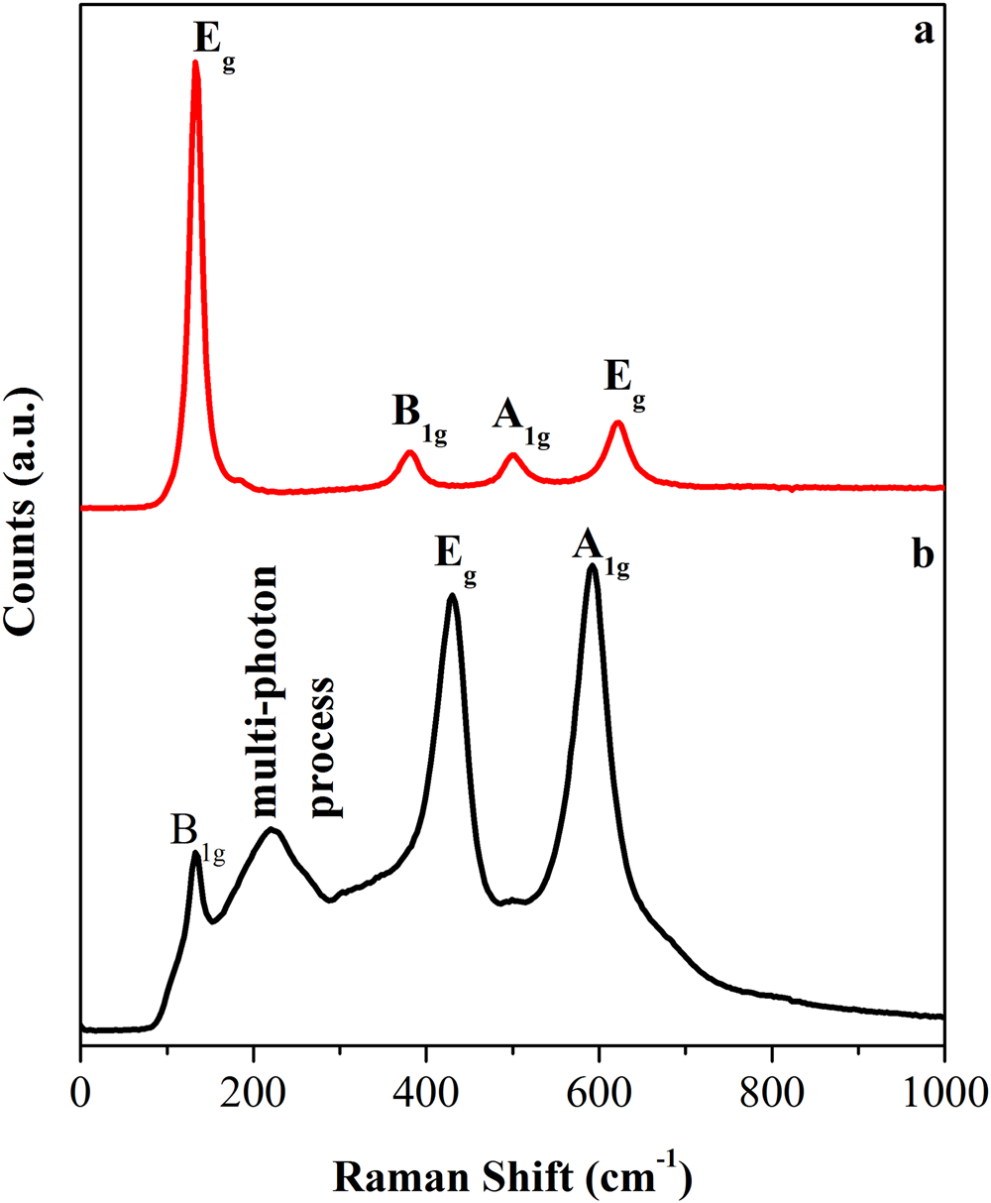
Raman spectra of (**a**) anatase, and (**b**) rutile TiO_2_.

X-ray diffraction (XRD) patterns in Fig. 1a and b represents the formation of phase pure tetragonal anatase (JCPDS: 89-4921; SG: I4_1_/amd) and rutile TiO_2_ (JCPDS: 89–6975; SG:P4_2_/mnm) synthesized by the solution combustion and polymerizable sol-gel approaches, respectively. The cell parameters of anatase were obtained to be a/b = 3.7794 Å and c = 9.4984 Å, and for rutile a/b = 4.5847 Å and c = 2.9557 Å (from refinement in Figure S1 in SI). The anatase showed a broader peak width than the rutile and the crystallite sizes of anatase and rutile calculated using Scherrer method from the (101) of anatase and (110) of rutile were 15.0 and 31.2 nm, respectively. As seen from the XRD, (101) of anatase and (110) of rutile are the highest intense peaks, thus it could be assumed that these are the mostly exposed facets of the materials for any catalytic reaction. Figure 2 also reveals the structural information from Raman spectroscopy. Four characteristic Raman active modes of anatase TiO_2_ with symmetries E_g_, B_1g_, A_1g_ and E_g_ were observed at 134, 382, 500, and 618 cm^−1^, respectively. These characteristic vibrational frequencies and their intensity ratios confirmed the phase pure anatase TiO_2_^54^. On the other hand, the rutile TiO_2_ exhibited characteristic stretching peaks at 140, 430, and 590 cm^−1^ that correspond to the symmetries of B_1g_, E_g_, and A_1g_, respectively^55^. In addition, another characteristic broad compound vibrational peak at 230 cm^−1^ aroused from the multiple phonon scattering processes was also clearly observed^56^. These XRD and Raman analyses confirm the formation of phase pure anatase and rutile TiO_2_.

**Figure 3:**
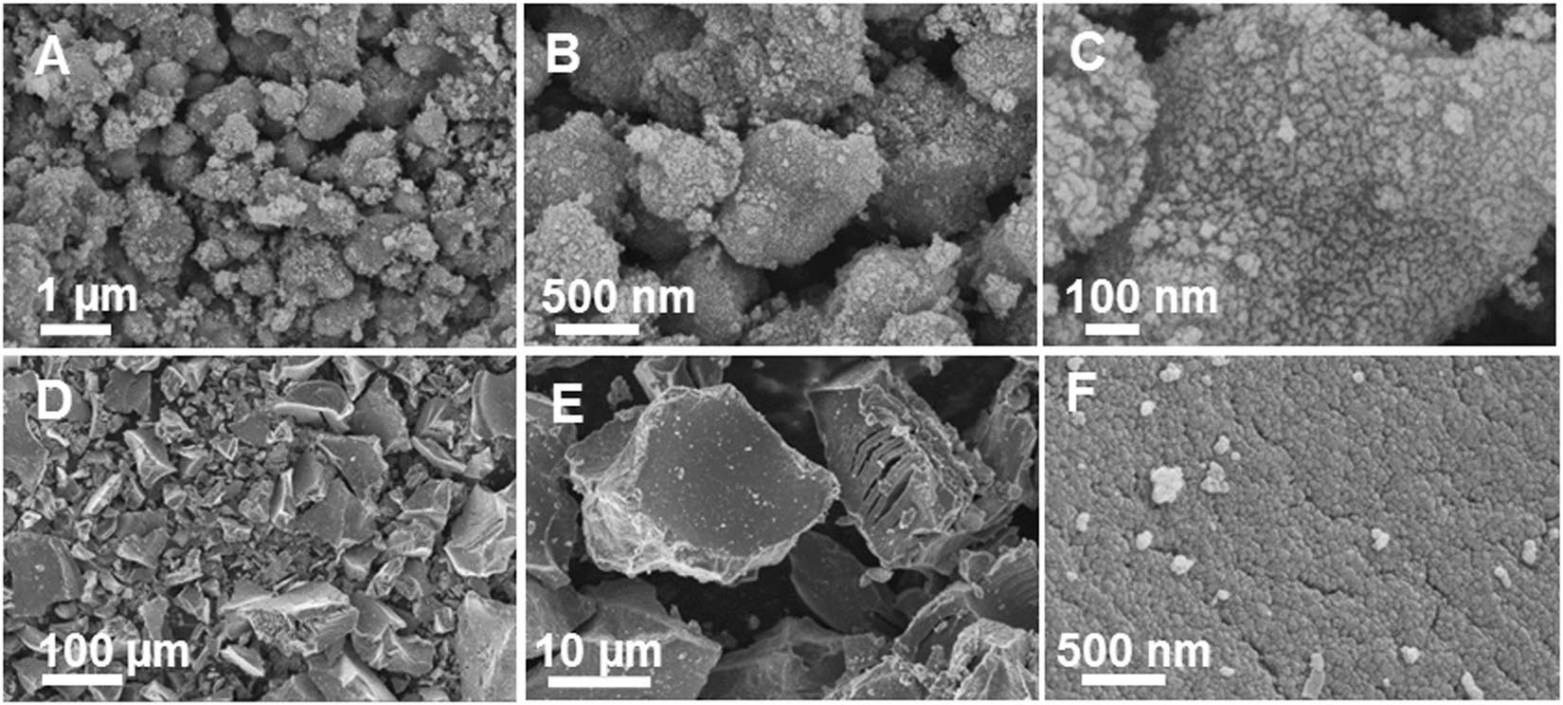
FE-SEM images of anatase (**A–C**), and rutile TiO_2_ (**D**–**F**) at different magnifications.

The size, shape and surface morphology of the synthesized anatase and rutile TiO_2_ materials were studied using FE-SEM and represented in Fig. 3. The anatase TiO_2_ synthesized by the combustion method was found to possess sub-micron particles that appear to be consisted of a few fused spheres. The high magnification imaging revealed the presence of dense nanocrystalline domains at the surface. The rutile TiO_2_ synthesized by the polymerizable sol-gel approach was found to be irregular rock like particles having large variation in size with the range of few to 100 microns. The high magnification imaging of these particles revealed the presence of nanocrystalline domains, however, the surface was found to be relatively smoother when compared to anatase.

**Figure 4:**
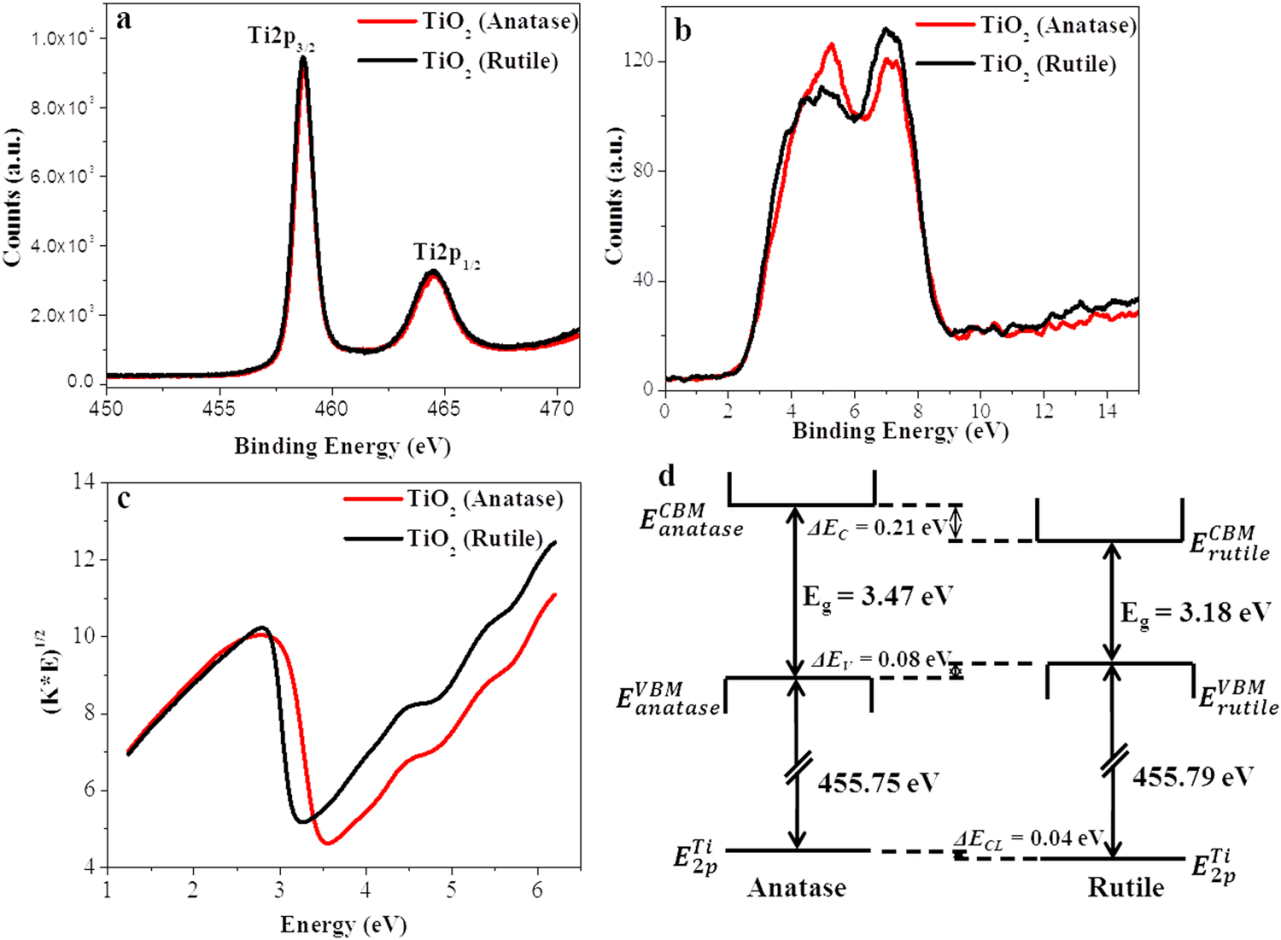
(**a**) The core level photoelectron spectra of Ti(2*p*), (**b**) valance band regions, (**c**) Tauc plot, and (**d**) schematic of XPS alignment with band gap obtained from Tauc plot of anatase and rutile TiO_2_. ΔE_V_ and ΔE_C_ are the valance band offsets and conduction band offsets, respectively and ΔE_CL_ core level offset between the Ti(2p_3/2_) core levels. E^VBM^ and E^CBM^ are the positions of the valance and conduction band, respectively and Eg is band gap obtained from Tauc plot.

The electronic information of the materials were obtained from the core level photoelectron spectra of Ti(2*p*) and valance band region of anatase and rutile TiO_2 _(plotted in Fig. 4a and b, respectively). The presence of Ti^4+^ is confirmed as Ti(2*p*_3/2_) and Ti(2*p*_1/2_) peaks were observed at 458.75 and 464.4 eV for anatase, and at 458.71 eV and 464.5 eV for rutile TiO_2_, respectively. The intensity of valance band spectra largely coincides with the density of state at each energy level and the valance band maxima is largely equal to binding energy when the intensity begins to increase. Apparently evident from Fig. 4b, the intensity of binding energy start increasing at 3.0 eV for anatase and 2.92 eV for rutile, concluding that the valance band maxima is lowered by 0.08 eV in anatase than that of rutile. The Tauc plot (Fig. 4c) was obtained from the diffuse reflectance spectra of anatase and rutile TiO_2_ (in Figure S2 in SI) and anatase was found to be having higher band gap (3.47 eV) than that of rutile (3.18 eV). The wide band gap of anatase and rutile TiO_2_ has also been reported previously^16, 57–59^. The valence and conducting bands of TiO_2_ are majorly constituted of O(2*p*) and Ti(3*d*), respectively. The different Ti-Ti and Ti-O distances in the anatase and rutile structure may lead to different electronic configuration, resulting in small variation in the band gap. The Ti 2*p*_3/2_ core line and the valence band maxima of anatase and rutile obtained directly from XPS were clubbed with the band gap obtained from Tauc plot and shown in Fig. 4d. As seen from the figure, the valence band maxima of rutile is slightly positioned above than that of anatase, however, the conduction band minima of anatase is 0.21 eV above than that of rutile. This concludes that the photo generated electrons in anatase are of higher energy and these along with protons from alcohol can efficiently reduce nitrobenzene.

**Figure 5:**
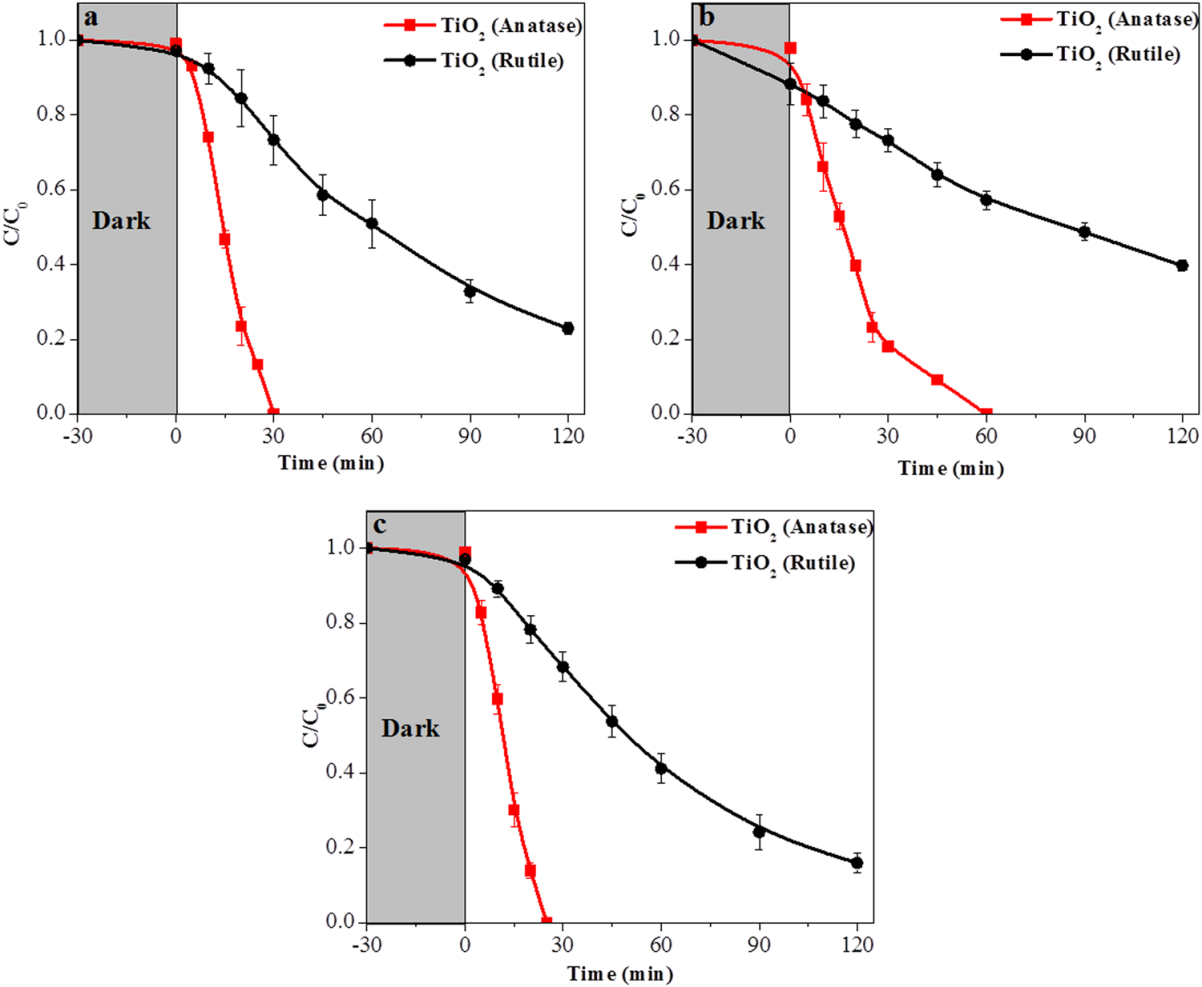
Photocatalytic reduction of (**a**) nitrobenzene, (**b**) 2-nitrotoluene, and (**c**) 2-fluoro-nitrobenzene under anaerobic conditions.

Both, anatase and rutile were explored for photocatalytic reduction of nitrobenzene under inert atmosphere in presence of methanol as the hole scavenger and plotted in Fig. 5a. In all the photocatalytic reduction experiments, the nitrobenzene was incubated with the catalyst in dark for 30 min to equilibrate the adsorption/desorption process before the light was turned on. As seen in Fig. 5a, the anatase TiO_2_ has reduced nitrobenzene completely to aniline within 30 min after the light was turned on, while the rutile even after 2 h of light exposure could only reduce 80% of nitrobenzene. Rate of nitrobenzene reduction over anatase was found to be 1.43 × 10^−2^ mmol L^−1^ g^−1^ s^−1^, whereas the rate was 1.78 × 10^−3^ mmol L^−1^ g^−1^ s^−1^ over rutile TiO_2_. The catalytic progress of the reaction was probed with GC (Figure S3 in SI). The nitrobenzene peak at 4.45 min gradually decreased with the formation of the aniline peak at 3.5 min. There was a formation of phenylhydroxylamine intermediate over anatase, however rutile TiO_2_ did not show this intermediate. The different mechanistic pathways could have been an influencing parameter in the catalytic activity of anatase and rutile TiO_2_. The catalytic reaction was also monitored by time dependent UV−visible spectroscopy (Figure S4 in the SI), where a gradual decrease in the intensity of the characteristic absorbance peak of nitrobenzene at 267 nm was observed, along with the development of new absorbance peaks at 230 and 280 nm corresponding to the formation of aniline. An attempt of nitrobenzene reduction over anatase and rutile TiO_2_ was also made in aerobic condition. The photoreduction profile under aerobic and anaerobic conditions over anatase was almost identical, whereas, the photoreduction under aerobic condition was highly suppressed over rutile (plotted in Figure S5 in SI). Further exploration was carried out to understand the effect of electron donating and withdrawing groups on the photocatalytic catalytic reduction of nitrobenzene over anatase and rutile TiO_2_. The anatase TiO_2_ again outperformed the rutile one in photoreduction of 2-nitrotoluene as well as 2-fluoro-nitrobenzene (Fig. 5b and c). No intermediate was detected for any of the photoreductions. Interestingly, over both anatase and rutile, the rate of the photoreduction followed the following trend: 2-fluoro-nitrobenzene >nitrobenzene >2-nitrotoluene (Figure S6 in the SI). We can assume that the photogenerated electrons from the conduction band of anatase or rutile may move downhill to the available antibonding orbitals of nitroarenes for reduction. As it was observed that the photogenerated electrons in anatase are of higher energy, they may easily reduce the nitroarenes compared to the rutile TiO_2_. Further, due to the presence of the electron donating group in the ring, this electron hopping may not be as facile as it could be due to the presence of an electron withdrawing group. Therefore, the rate of photoreduction was found higher over 2-fluoro-nitrobenzene than that of 2-nitrotoluene.

**Figure 6:**
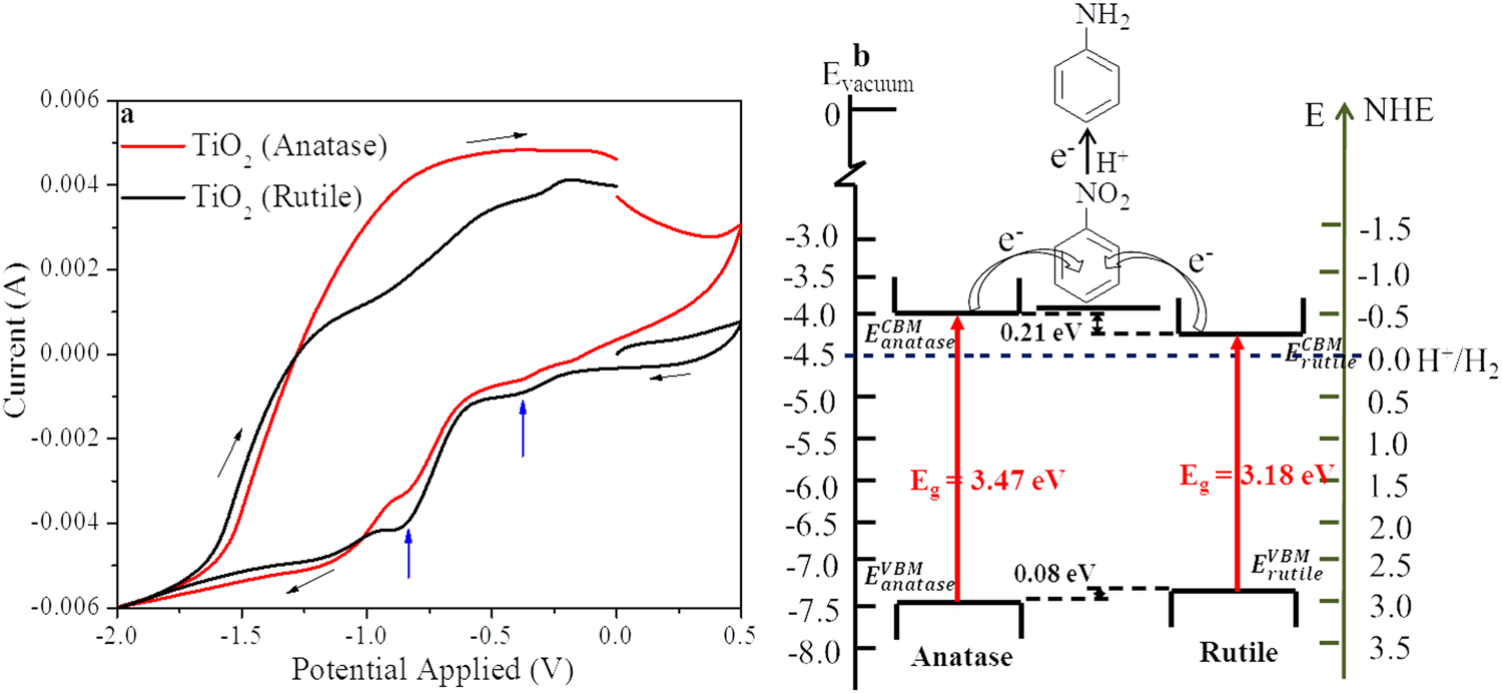
(**a**) Cyclic voltammetric study for electrochemical reduction of nitrobenzene over anatase, and rutile TiO_2_, and (**b**) Schematic illustration of energetics of nitrobenzene reduction over anatase, and rutile TiO_2_.

To probe it further, electrochemical reduction of nitrobenzene was carried out on anatase and rutile TiO_2_ and plotted in Fig. 6a. The first voltammetric wave at −0.38 V vs. Ag/AgCl (−0.16 V vs. NHE) was observed over the materials followed by a second peak at −0.80 V vs. Ag/AgCl (−0.58 V vs. NHE) (Figure S7a–c in SI shows the CV with the electrolyte and with and without nitrobenzene by using anatase and rutile TiO_2_ electrode shows the CV with graphite electrode)^60^. It may be concluded that the electrochemical reduction of nitrobenzene happens in two steps over both the catalysts^61–63^, and there is no difference in electro catalytic reduction efficiency over the two catalysts as the reduction potentials appear in the same position. It must be noted that the reduction potentials are negative than the hydrogen reduction potential and are very close to the conduction band minima of anatase and rutile. The complete reduction potential of nitrobenzene obtained from CV is aligned with the valence and conduction band energetics of anatase and rutile TiO_2_ obtained through XPS and Tauc plot (Fig. 6b). In the figure, we have represented the valence and conduction band energetics on the absolute vacuum scale coupled with the normal hydrogen electrode (NHE). It is known that electrons can only be transferred between those energetic states in the semiconductor and the reactant that are approximately at the same energy level. As it is evident from the figure that the conduction band energy minima of anatase (−0.47 V) is slightly positioned above than that of rutile (−0.26 V), the electron movement from the conduction band of anatase to nitrobenzene would be more facile. The electrochemical reduction of the substituted nitroarenes revealed the reduction potentials of 2-nitrotoluene and 2-fluoro-nitrobenzene to be −0.66 and −0.53 V (vs. NHE), respectively (Figure S7c, in SI). As the reduction potential of electron donating 2-nitrotoluene is placed above that of nitrobenzene (−0.58 V vs. NHE), its reduction is more challenging, which has also been observed from the photocatalytic experiments. On the contrary, the reduction potential of electron withdrawing 2-fluoro-nitrobenzene being placed in a favorable position, its reduction is found to be facile.

**Figure 7:**
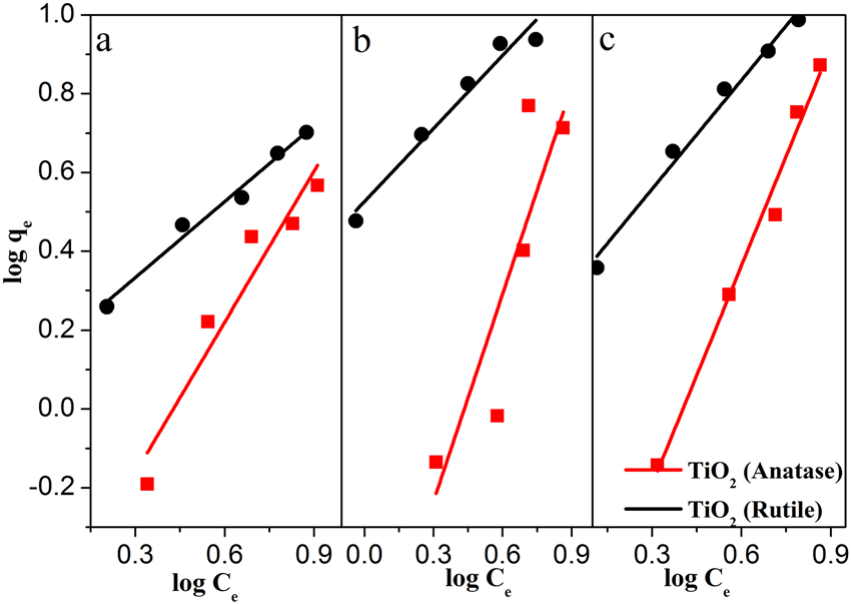
Freundlich adsorption isotherm model for (**a**) nitrobenzene, (**b**) 2-nitrotoluene, and (**c**) 2-fluoro-nitrobenzene anatase and rutile TiO_2_. The equilibrium concentration (q_e_) of nitroarenes was calculated by using following equation: q_e_ = (C_0_−C_e_)V/W, where C_0_ and C_e_ are initial and final concentrations of nitroarenes (mg/L), V is amount of solution (L) and W is weight of catalyst (g).

**Figure 8:**
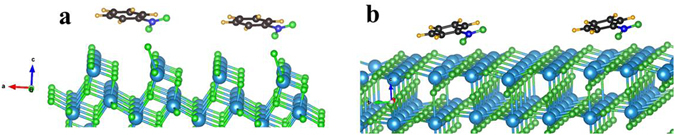
Adsorption of nitrobenzene over (**a**) constructed (101) anatase, and (**b**) (110) rutile surfaces of TiO_2_.

The higher position of conduction electron may not be the only reason for higher catalytic efficiency of anatase TiO_2_ over rutile, but adsorption can also play the pivotal role as it is commonly an essential first step in any heterogeneous photocatalysis. Therefore, we investigated the dark adsorption efficiency of the three nitroarenes over anatase and rutile TiO_2_. The amount of nitroarenes adsorbed over unit weight of TiO_2_ (q_e_/mg g^−1^) is plotted against the amount of nitroarenes remaining in the bulk solution at equilibrium (C_e_/mg L^−1^) and was attempted to fit with Langmuir and Freundlich isotherms. The experimental observations indicates that the nitroarenes could get adsorbed on to anatase and rutile TiO_2_ according to the Freundlich isotherm model (Fig. 7) and the value of regression coefficient for the adsorption model was closed to unity (Table 1). To our surprise, all the nitroarenes were better adsorbed over rutile than anatase TiO_2_ and the relative adsorption capacity was higher almost by an order of magnitude (vide Freundlich exponent, K_F_ in Table 1). Independently, we have also performed the nitroarenes adsorption from First-principles DFT calculations. As evident from XRD, (101) and (110) are the most exposed facets of anatase and rutile, respectively, nitroarenes were adsorbed over these constructed surfaces (Fig. 8) and the computed adsorption energies are given in Table 2. For all the nitroarenes the adsorption energy minima are almost twice over rutile (110) compared to anatase (101). This is corroborating our experimental adsorption results. Therefore, it can be concluded that though nitroarenes adsorption over rutile is higher but it does not play any favorable role in their photoreduction. Interestingly, the optimum distances of nitrobenzene over anatase (101) and rutile (110) corresponding to the minimal energy were found to be 2.237 Å and 2.917 Å, respectively. The closer proximity of nitrobenzene to the (101) of anatase may facilitate the conduction electron hopping from the surface to the adsorbate.

**Table 1:** The relative adsorption capacity (K_F_) was obtained from Freundlich adsorption isotherm, log q_e_ = log K_F_ + 1/n (log C_e_), where q_e_ is the equilibrium concentration of nitroarenes, C_e_ is final concentrations of nitroarenes, n is intensity of adsorption, and R^2^ is the regression coefficient.

Nitroarenes	K_F_ (mg^1-1/n^ g^−1^ L^1/n^)		n		R^2^	
	Anatase	Rutile	Anatase	Rutile	Anatase	Rutile
Nitrobenzene	0.285	1.379	0.783	1.554	0.963	0.992
2-nitrotoluene	0.174	3.356	0.570	1.617	0.974	0.982
2-fluoro- nitrobenzene	0.179	1.912	0.541	1.085	0.993	0.993

**Table 2:** The computed adsorption energies of nitroarenes over (101) of anatase, and (110) of rutile TiO_2_. The adsorption energies correspond to the stabilization energy of the nitroparenes over the constructed surface.

Nitroarenes	Adsorption Energy (meV)	
	Anatase (101)	Rutile (110)
Nitrobenzene	257	383
2-nitrotoluene	194	409
2-fluoro- nitrobenzene	259	502

The stepwise photocatalytic reduction mechanism could be described as follows:

1 $$Ti{O}_{2}\,\to {e}_{CB}^{-}+{h}_{VB}^{+}$$

2 $$C{H}_{3}OH+{h}_{VB}^{+\,}\to HCHO+{H}^{+}$$

3 $$Ar-N{O}_{2}+6{H}^{+}+6{e}_{CB}^{-}\to Ar-N{H}_{2}+2{H}_{2}O$$

4 $${e}_{CB}^{-}+{h}_{VB}^{-}\to Recombination$$

**Figure 9:**
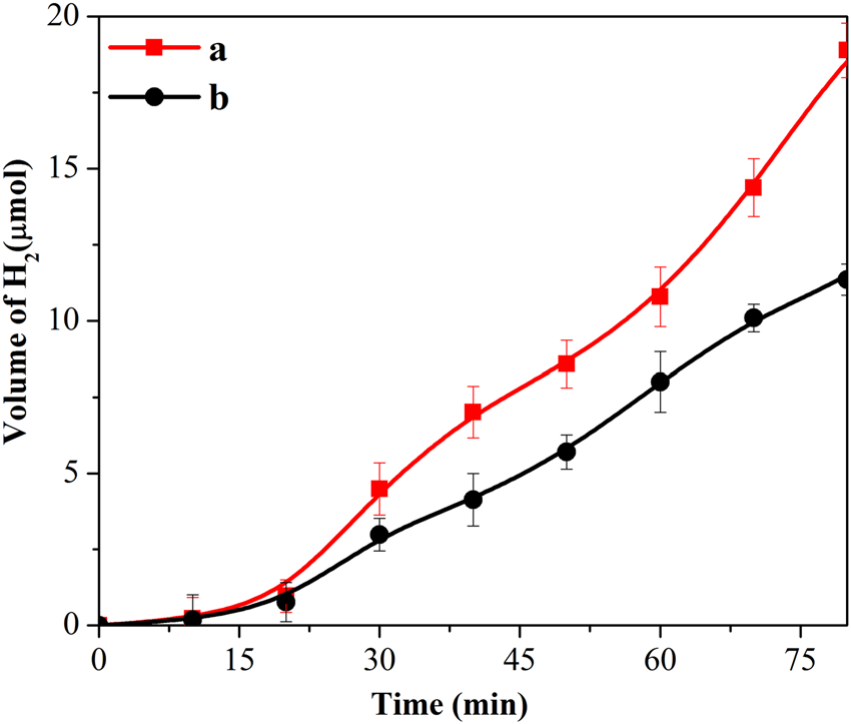
Photocatalytic hydrogen production over (**a**) anatase, and (**b**) rutile TiO_2_.

**Figure 10:**
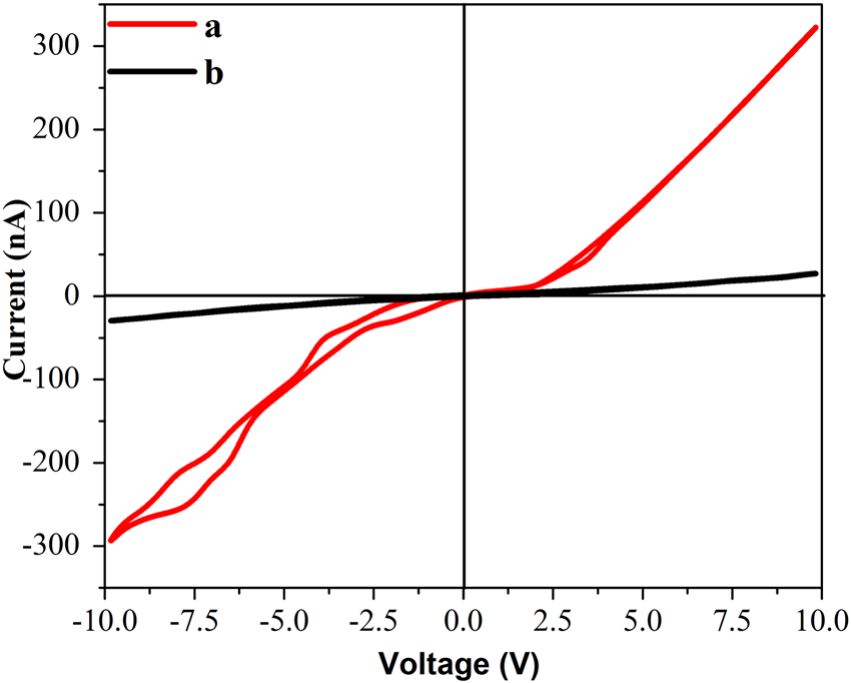
The current generation as a function of applied voltage under UV light exposure over (**a**) anatase, and (**b**) rutile TiO_2_.

Methanol was used in the reaction medium to minimize the step 4. Methanol can act as a hole scavenger and upon oxidation produces protons, which in turn reduce nitroarenes along with the photogenerated electrons (step 3). Therefore, the efficiency of electron and proton generation (steps 1 and 2) over the catalysts may also be the determining factors in nitroarenes reduction. The photo production of hydrogen over anatase and rutile was carried out to compare the proton generation efficiencies over both the materials. Figure 9 compares the hydrogen production, which reveals that initially there was no difference, however with advent of time anatase yielded only ~64% higher amount of hydrogen than rutile. It must be noted that the rate of nitrobenzene reduction over anatase was higher by an order of magnitude than that of rutile, which indicates that the proton generation (step 2) is not directly influencing the efficiency of nitrobenzene reduction. On the other hand, different electrical conductivity behavior of the phases under light exposure could determine the availability of the photogenerated electrons. The current generation as a function of applied voltage under UV light exposure was studied with both the catalysts and the results are plotted in Fig. 10. It is evident from the figure that the current observed in anatase is significantly higher compared to rutile. The higher current generation in anatase could be due to (i) more electron-hole generation, and/or (ii) lesser electron-hole recombination. To probe this further, we have additionally performed the photoluminescence (PL) spectroscopy with both the catalysts and the spectra (plotted in Figure S8) shows higher peak intensity of rutile than that of anatase TiO_2_. Generally, the lower PL intensity signifies lower electron-hole recombination. The observation from PL can claim lower electron-hole recombination in anatase than that in rutile TiO_2_. The higher number of photogenerated electrons in the conduction band of anatase may facilitate rapid nitroarenes reduction.

## Conclusion

The phase pure anatase and rutile TiO_2_ were produced by solution combustion and polymerizable sol-gel syntheses, respectively. Both the catalysts were studied for nitroarenes reduction and anatase was found to be outperforming the rutile. The phase dependent nitroarenes reduction has been investigated through (i) energetics of conduction band minima, (ii) adsorption efficiency, (iii) reduction potential of adsorbed nitroarenes, (iv) proton generation, and (v) photo generation of electrons over both anatase and rutile TiO_2_. The proton generation and reduction potential of nitrobenzene over both the catalysts was comparable. The nitroarenes adsorption over rutile was an order of magnitude higher than anatase. Thus the proton generation and adsorption did not play any significant role in the higher reduction efficiency of anatase. Interestingly, the position of conduction band minima of anatase was found to be 0.21 eV higher than that of rutile and also it was observed that the photo generated electrons in the conduction band of anatase are more in number than rutile. The greater flux of high energy electrons in anatase therefore favored the nitroarene reduction than rutile. This study may pave the path on exploring the energetics of a structure dependent photocatalytic reaction.

## Electronic supplementary material

Supplementary Data
