# Supplementary material for: Structure sensitive photocatalytic reduction of nitroarenes over TiO2
Source: Sci Rep. 2017 Aug 18;7:8783. doi: 10.1038/s41598-017-08599-2 (PMC5562743; doi:10.1038/s41598-017-08599-2)
Supplement: Supplementary file 1 — Supplementary Data [file 41598_2017_8599_MOESM1_ESM.pdf]

Structure sensitive photocatalytic reduction of nitroarenes over TiO_2

Swapna Challagulla[1], Kartick Tarafder[2], Ramakrishnan Ganesan[1]*, Sounak Roy[1]*

1 Department of Chemistry, Birla Institute of Technology and Science (BITS) Pilani, Hyderabad Campus, Jawahar Nagar, Shameerpet Mandal, Hyderabad-500078, India

2. Department of Physics, National Institute of Technology Karnataka, Surathkal, Mangalore - 575 025 Karnataka, India.

Figure: S1

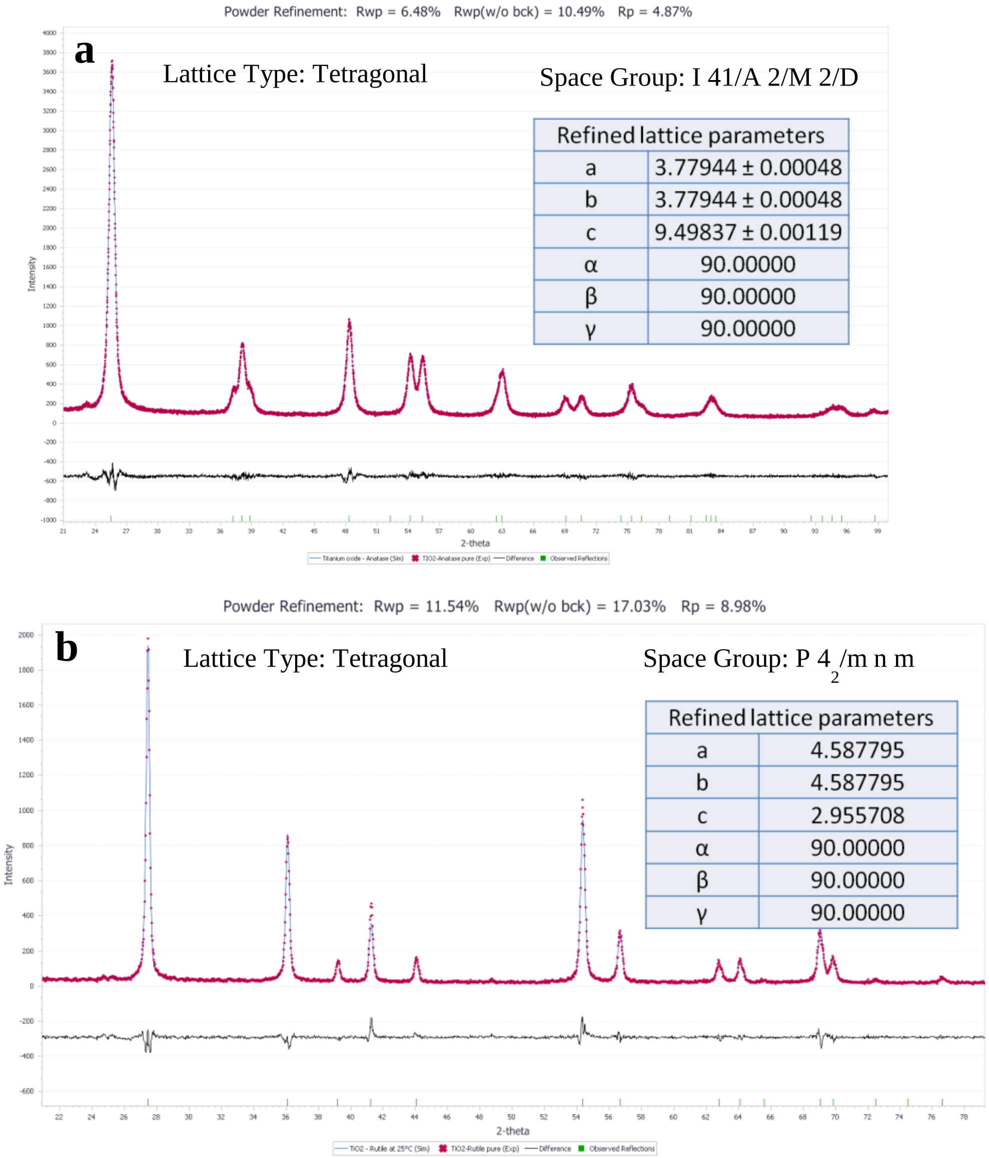

Figure S1: Refinement of a) anatase, and b) rutile TiO_2.

Figure: S2

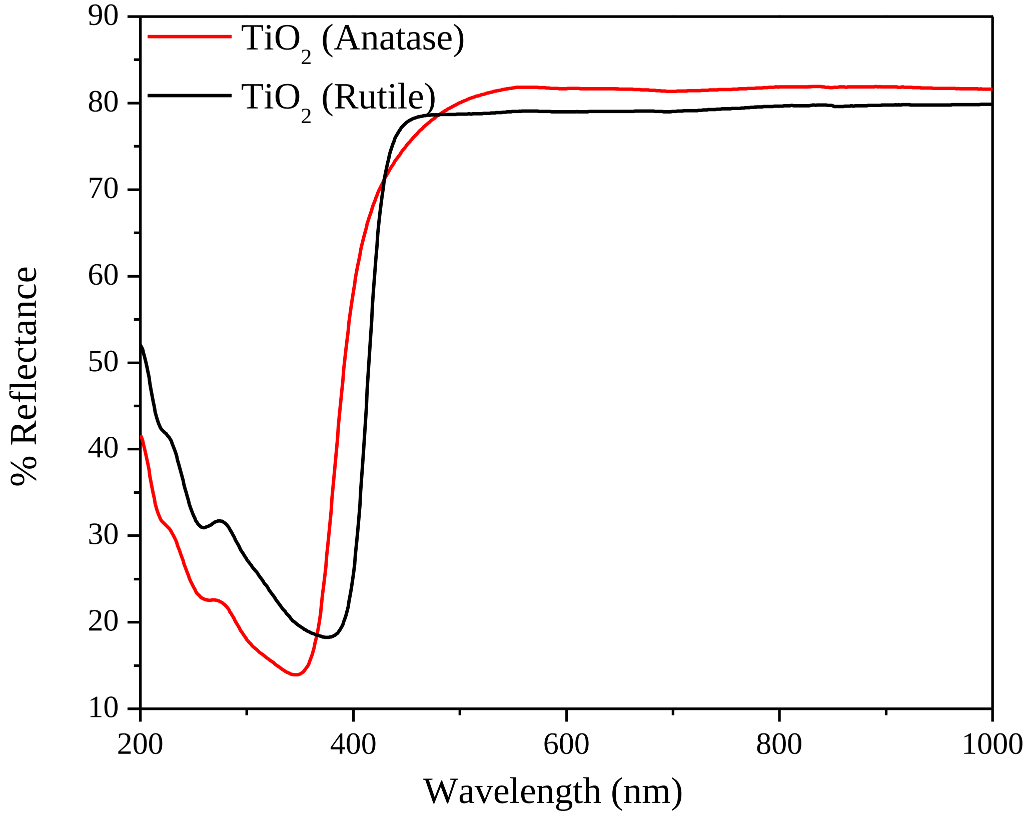

Figure S2: The diffuse reflectance spectra of anatase, and rutile TiO_2.

Figure: S3

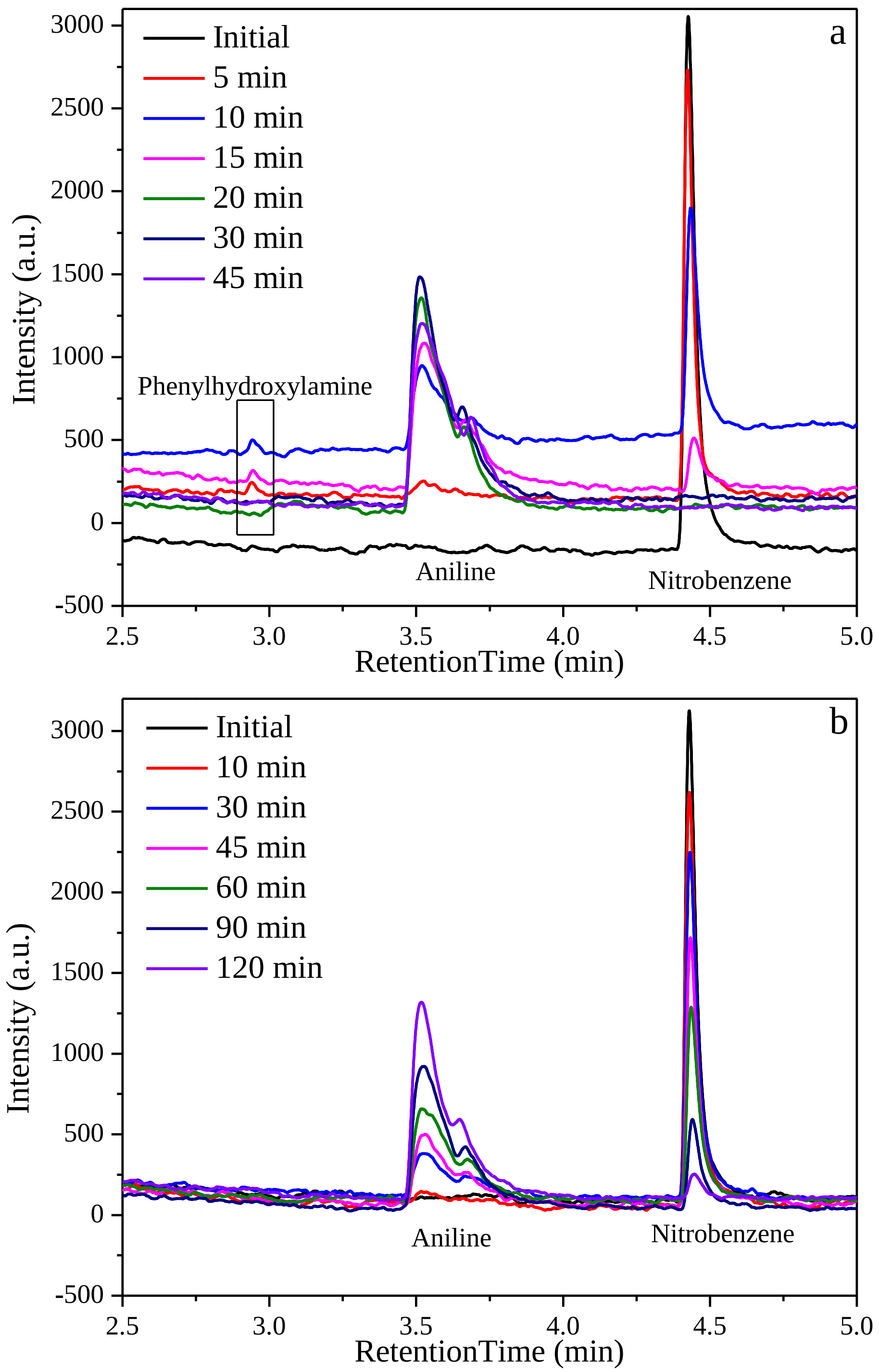

Figure S3: GC profile of nitrobenzene over a) anatase, and b) rutile TiO_2. The nitrobenzene peak at 4.45 min decreased with the progress of the reaction and the formation of the aniline peak at 3.5 min was observed. The anatase TiO_2 (a) shows a peak of phenylhydroxylamine at ~2.95 min with anatase TiO_2, which is absent in case of rutile (b).

Figure: S4

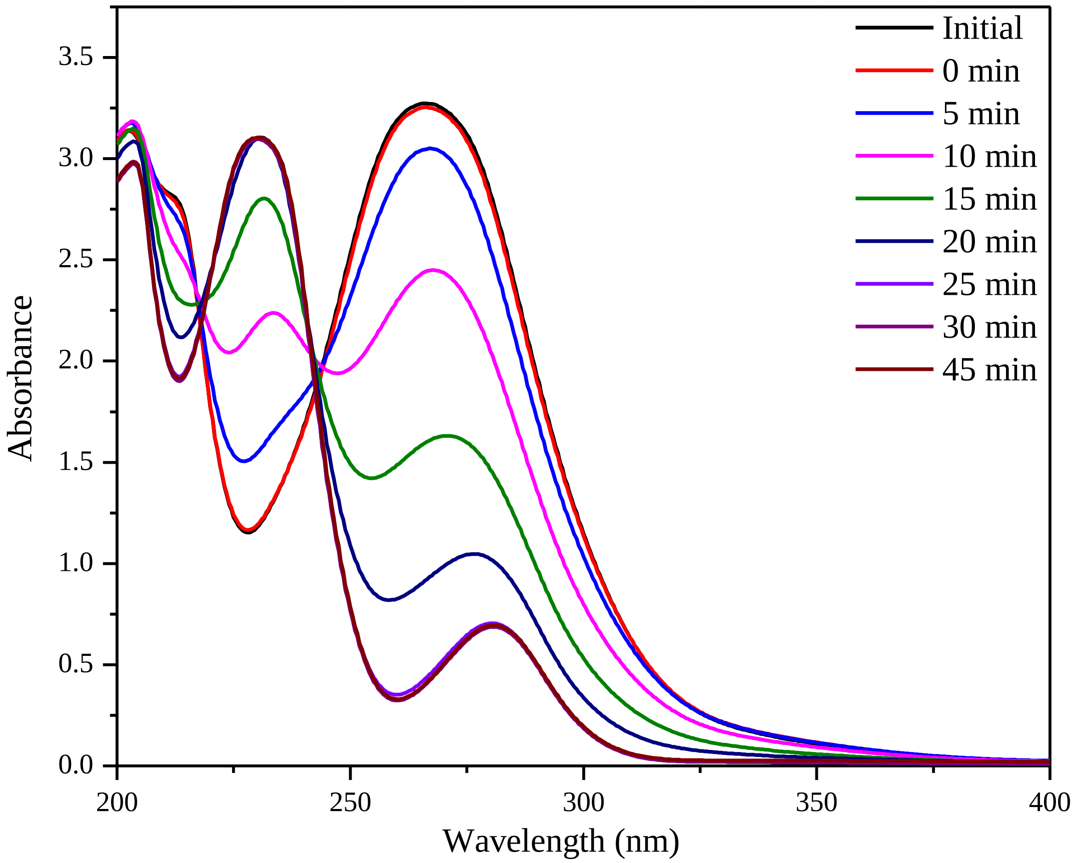

Figure S4: Probing photocatalytic reduction of nitrobenzene over anatase TiO_2 with time dependent UV−Visible spectroscopy. The characteristic absorbance peak of nitrobenzene at 267 nm was gradually decreased, and the new absorbance peaks at 230 and 280 nm corresponding to the aniline was developed.

Figure: S5

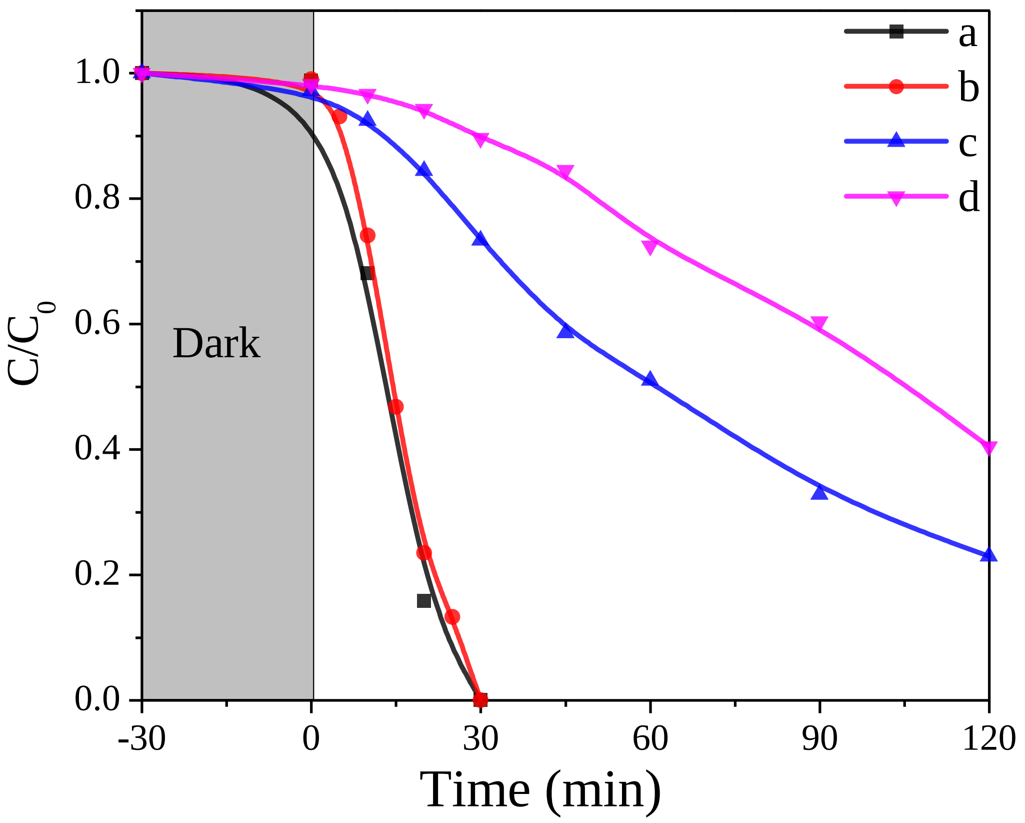

Figure S5: Photocatalytic reduction of nitrobenzene over anatase in a) anaerobic, b) aerobic, and rutile TiO_2 in c) anaerobic, and d) aerobic conditions.

Figure: S6

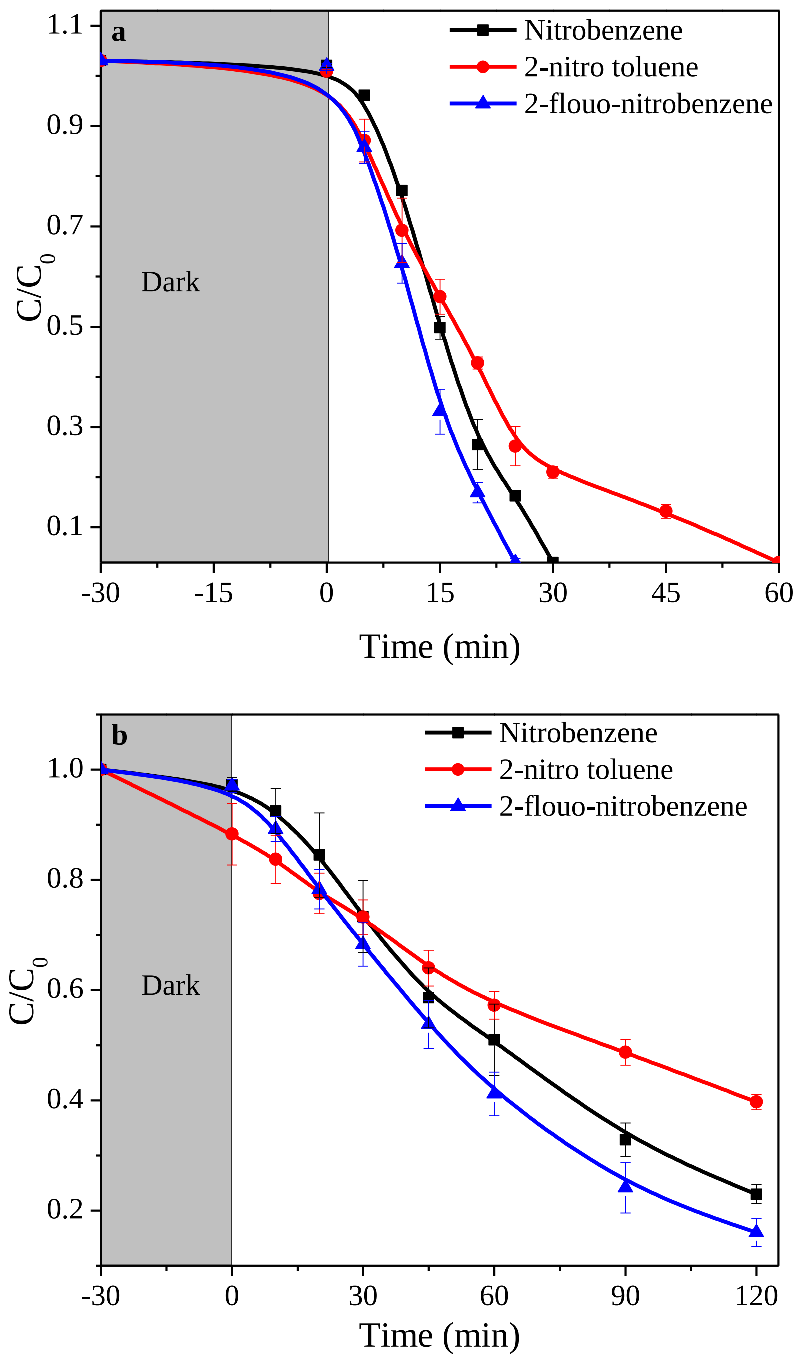

Figure S6: Photocatalytic reduction of nitrobenzene, 2-nitrotoluene and 2-fluoro-nitrobenzene over a) anatase, and b) rutile TiO_2.

Figure: S7

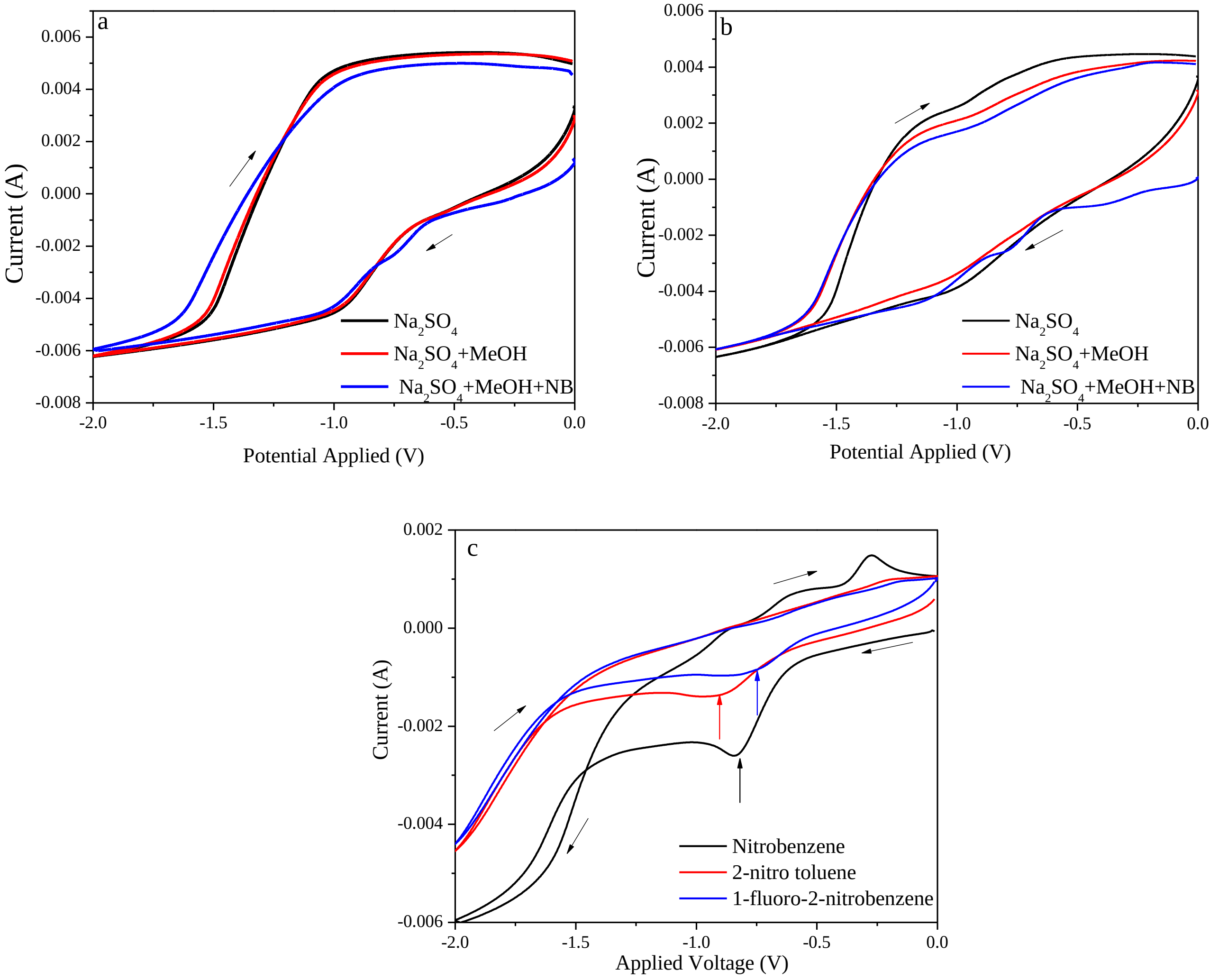

Figure S7: Electrochemical reduction of nitrobenzene with different electrolytes over a) anatase, and b) rutile TiO_2. c) Nitrobenzene, 2-nitrotoluene, and 1-fluoro-2-nitrobenzene reduction over graphite electrode.

Figure S8

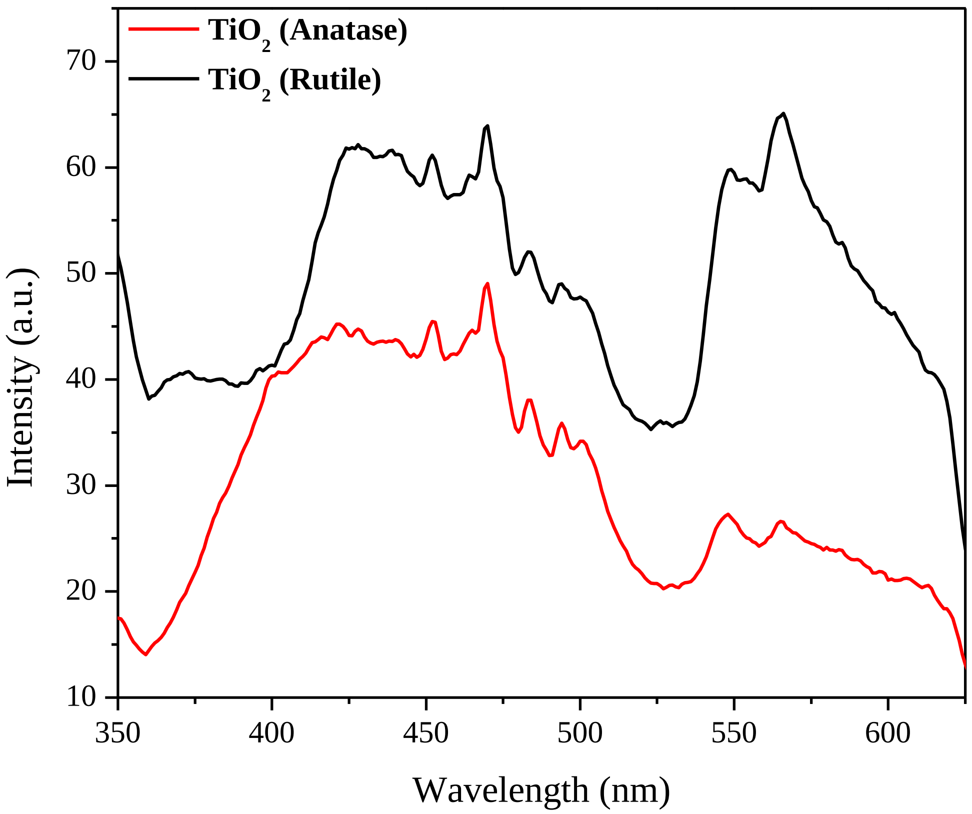

Figure S8: Photoluminescence spectra of anatase and rutile TiO_2 after excitation at 330 nm.
